# Conjunctival Marginal Zone Lymphoma in a Patient With Neurofibromatosis Type 1: A Case Report

**DOI:** 10.7759/cureus.49491

**Published:** 2023-11-27

**Authors:** Chrysoula Florou, Evaggelia Aissopou, Evangelia Papakonstantinou, Ilias Georgalas, Konstantinos Droutsas

**Affiliations:** 1 Department of Ophthalmology, National and Kapodistrian University of Athens, General Hospital "G. Gennimatas", Athens, GRC; 2 Department of Ophthalmology, Macula Center, Athens, GRC

**Keywords:** neurofibromatosis type 1, neurofibromin, non-hodgkin lymphoma, extranodal marginal zone lymphoma, chemo-immunotherapy

## Abstract

We present a case of painless bulbar conjunctival mass due to B-cell non-Hodgkin lymphoma (NHL), without systemic involvement, in a 76-year-old man. Following an excision biopsy, histopathologic examination and immunohistochemistry confirmed the diagnosis, prompting a referral for hemato-oncological assessment. The patient underwent comprehensive laboratory and imaging scans, subsequently receiving combined chemo-immunotherapy that resulted in complete remission to date. This case is reported as it is crucial to recognize that a conjunctival insult might emerge in neurofibromatosis type 1 (NF1) patients.

## Introduction

Neurofibromatosis type 1 (NF1), commonly referred to as von-Recklinghausen disease or peripheral neurofibromatosis, stands out as one of the frequently encountered autosomal dominant conditions in clinical practice [1], affecting approximately one in 2,500 births [2]. The diagnosis is mainly based on clinical criteria including café au lait macules, neurofibromas, freckling on the axillary and/or groin areas, Lisch nodules (iris hamartomas), bone dysplasia, and optic pathway gliomas [3]. NF1 patients are at increased risk of developing malignancies because of the loss of function of the tumor-suppressor gene that encodes neurofibromin, a crucial protein in the Ras/mitogen-activated protein kinase (MAPK) pathway [4]. Among the nonhematologic malignancies frequently observed in NF1 are soft tissue sarcomas, specifically malignant peripheral nerve sheath tumors or rhabdomyosarcomas, along with gastrointestinal stromal tumors, glomus tumors, and those affecting the central nervous system [5,6]. Although NF1 patients have a 5- to 10-fold increase in relative risk of non-Hodgkin lymphoma (NHL) [7,8], there have been no reported cases of conjunctival involvement in terms of NHL so far.

In this report, we describe the first documented case at our institution of an elderly NF1 patient who experienced an extranodal marginal zone B-cell lymphoma (EMZL) with plasmacytoid differentiation and a monoclonal IgM paraprotein of 1160 mg/dl, free of any systemic involvement.

## Case presentation

**Figure 1:**
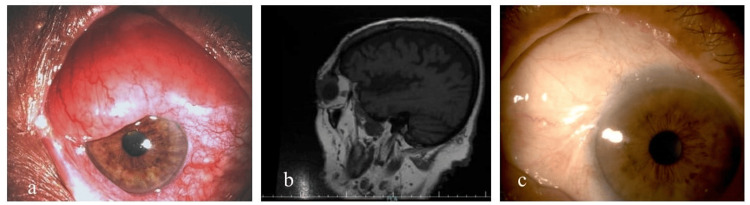
(a) Slit lamp photograph of the left eye of our NF1 patient with a conjunctival EMZL, (b) brain MRI with paramagnetic contrast before treatment, (c) slit lamp photograph of the left eye after excision of the mass

A male aged 76, with a medical history of NF1 since childhood, emerged with a painless bulbar conjunctival mass in his left eye that had developed over the past six months. Until this occurrence, the patient had led a relatively healthy life with only mild effects from NF1, and no systemic neoplasia was reported. Upon examination using slit lamp biomicroscopy, a mass originating from the upper nasal bulbar conjunctiva was observed, extending over the adjacent limbus and peripheral cornea, accompanied by diffuse hyperemia (Figure 1a). The anterior chamber showed normal depth and was quiet, intraocular applanation tonometry was within the normal range, and fundoscopy revealed no pathological findings. However, his visual acuity was compromised at 4/10 due to an advanced nuclear cataract. The patient underwent an orbit and brain MRI with paramagnetic contrast, which confirmed a homogenous, unifocal mass with smooth ocular margins. Importantly, the imaging ruled out the intraneural spread of the tumor or invasion into extraocular muscles (Figure 1b). Since no imaging method could provide a definitive differential diagnosis, the decision was made for a surgical excisional biopsy. A meticulous dissection of the conjunctiva was employed to separate the tumor, followed by a wide excision with some margins, extending to the equator. A perilimbal area was then covered with an amniotic membrane patch, secured with fibrin glue (TISSEEL) and a 10.0 nylon suture (Figure 1c).

Following resection, the lesion was mapped, marked, fixed in formalin, and sent for histological and immunophenotypical analysis. The results confirmed a low-grade B-cell NHL consistent with conjunctival EMZL with plasmacytoid differentiation, accompanied by a monoclonal IgM paraprotein of 1160 mg/dl. Postoperatively, the patient received local antibiotic coverage following the histopathological results. A referral to hemato-oncology was planned for staging and disease extent evaluation. A hemato-oncological referral was planned for staging and evaluation of the disease extent at that point. The patient underwent combined chemo-immunotherapy, including six cycles of rituximab and chlorambucil, followed by an additional six cycles of chlorambucil (10 mg orally for 10 days per month). Radiation therapy was deemed unnecessary. Subsequent hematological and imaging controls indicated remission that persists to the present day. The patient provided written informed consent for the publication of his case and accompanying images.

## Discussion

We present the case of a 76-year-old man with NF1, diagnosed with conjunctival B-cell NHL compatible with EMZL with plasmacytoid differentiation and a monoclonal IgM paraprotein of 1160 mg/dl, without any systemic involvement. The specific risk for malignancy in NF1 patients remains unknown, with estimates ranging from 3% to 48% and up to a fourfold increased risk compared to the general population [5]. Moreover, in hematologic malignancies, large population-based pediatric studies have reported an elevated risk of NHL among children with NF1 [9], although conjunctival NHL insult without systemic disease in NF1 patients has not been reported thus far.

NHLs are a diverse group of lymphoproliferative diseases typically affecting nodal and extranodal sites, displaying various nonspecific clinical features, and primarily affecting B lymphocytes. The heightened risk of lymphoid malignancies in NF1 is perplexing, given that these malignancies do not primarily include cells derived from the neural crest [8]. While convincing evidence for this association is still lacking, research suggests that the murine homologs of two genes embedded within the NF1 gene, EVI2A and EVI2B, are implicated in leukemogenesis in mice and are expressed in human white blood cells and bone marrow [10]. Further genetic mapping is required to explore this theory.

Tumorigenesis in NF1 includes several mechanisms, one of which is the deregulation of Ras signaling, a short region within neurofibromin containing a guanosine triphosphatase activating protein domain that regulates Ras/raf/ERK signaling and thus cellular growth and differentiation [5]. Patients with NF1 already possess a constitutional mutation in one allele of the NF1 gene, and if the other allele is silenced by a somatic mutation, the control of p21 Ras is absent contributing to malignancy (second-hit theory) [11]. Ras signaling is found to be involved in 30% of all cancer types. An additional study by Shapira et al. [12], on the other hand, revealed that neurofibromin is a complex protein with multiple functions that are not fully understood, playing a role in the regulation of cellular proliferation. In their experiment, Ras inhibitors used in cells with NF1 mutations did not induce apoptosis, contrary to expectations if the apoptotic pathway were solely Ras-dependent. In addition, neurofibromin seems to be involved in cAMP production. Dasgupta et al. [13] demonstrated that NF1 mutations in mice lead to reduced cAMP production and, consequently, limited control of cellular growth. Somatic NF1 gene aberrations have also been documented in tumors derived from non-NF1 patients, further highlighting the role of neurofibromin [14].

The conjunctiva, housing specialized lymphoid tissue, functions as an antigen barrier within the mucosa-associated lymphoid tissue (MALT) system [15]. Conjunctival lymphomas predominantly belong to the category of extranodal NHLs, with the primary subtype being EMZL of MALT type (66%) [16]. This type is believed to originate from neoplastically transformed marginal zone cells in reactive follicles [17]. Clinically, EMZL typically exhibits an indolent course and is recognized as a disease primarily affecting the elderly [18]. The recognized standard for diagnosing conjunctival lymphomas is through an incisional biopsy, allowing for histopathological and cytological examination. However, in our case, we chose a surgical excisional biopsy due to the small size of the mass, its well-circumscribed nature, and the absence of systemic lymphoma indications.

Ocular EMZL typically presents with a restricted number of highly indicative signs, such as salmon patches, insidious painless development, orbital mass, irritation, redness, and inflammation-like symptoms. Most commonly, these manifestations are unilateral, with bilateral presentation occurring in only 13% of cases. Regarding the differential diagnosis of this disease entity, chronic conjunctivitis, reactive or atypical lymphoid hyperplasia, atypical pterygium, episcleritis, IgG4-related disease, orbital inflammation, and orbital pseudotumor should be considered. The primary limitation of the manuscript lies in the rarity of the two described diseases, preventing the accumulation of additional cases necessary to establish a conclusive etiological correlation between them.

## Conclusions

This case is noteworthy, emphasizing the significance of considering lymphoma diagnosis in NF1 patients presenting with an orbital mass, followed by a biopsy to definitively confirm the diagnosis. While it is plausible that NF1 and conjunctival EMZL may have been manifested as distinct entities in our patient, it is crucial to acknowledge NF1 as a risk factor for such malignancies. This recognition is pivotal for implementing appropriate diagnostic and therapeutic strategies.
